# A gene therapy strategy for Deoxyhypusine Synthase (DHPS) syndrome

**DOI:** 10.21203/rs.3.rs-9621764/v1

**Published:** 2026-05-21

**Authors:** Carolina Nunes Santo, Mollie R. Shinkle, Rebeca Blanch, Angela Dias, Chengzu Long, Olin Konishi, Qiaoyan Yang, Orrin Devinsky, Juliana Laze, Teresa L. Mastracci, Alysson R. Muotri

**Affiliations:** University of California San Diego; Indiana University Indianapolis; University of California San Diego; University of California San Diego; NYU Grossman School of Medicine; NYU Grossman School of Medicine; NYU Grossman School of Medicine; NYU Grossman School of Medicine; NYU Grossman School of Medicine; Indiana University Indianapolis; University of California San Diego

## Abstract

Deoxyhypusine synthase (DHPS) syndrome is a rare, autosomal recessive neurodevelopmental disorder caused by biallelic pathogenic variants in the *DHPS* gene, which encodes deoxyhypusine synthase. This enzyme is essential for the post-translational hypusination of eukaryotic translation initiation factor 5A (eIF5A), a modification crucial for cell viability, protein synthesis, and neuronal development. Patients with DHPS deficiency typically present with global developmental delays, intellectual disabilities, speech and motor impairments, seizures, and various dysmorphic features. Molecular studies show that *DHPS* mutations disrupt eIF5A hypusination, impairing translation elongation and cellular homeostasis. Animal and cellular models have confirmed the neurotoxic effects of impaired hypusination. Although no targeted therapy is available, advances in understanding the molecular basis of the disorder have enabled translational research, including modulation of polyamine metabolism. Here, we describe the development of a gene therapy strategy to deliver *DHPS* cDNA to mutant human brain cells in cortical organoids derived from patient stem cells, successfully restoring hypusination. This approach also improved survival in a mouse model of DHPS deficiency, highlighting the potential of rescuing DHPS expression as a treatment.

## Introduction

Deoxyhypusine synthase (DHPS) plays a crucial role in a unique and essential post-translational modification called hypusination^1^. This two-step enzymatic process modifies a single lysine residue in eukaryotic initiation factor 5A (eIF5A), producing the hypusinated form, eIF5A^2^. This modification uses the polyamine spermidine as a cofactor to activate eIF5A, a highly conserved protein essential for mRNA translation and stability^1^. Germline deletion of *Dhps* is embryonically lethal by day 4.5 in mice^3^. Given the vital role of eIF5A in regulating protein synthesis and cellular proliferation, DHPS function is necessary for the development of many organs, including the brain, in mice^1^. Therefore, it was surprising when the rare monogenic disease DHPS deficiency was found to be caused by mutations in the *DHPS* gene^1^.

DHPS deficiency was first identified in five patients, characterized by epilepsy, global developmental delay, hypotonia, speech and motor impairments, and craniofacial dysmorphisms^1^. Affected individuals inherited biallelic *DHPS* mutations; each with a missense mutation in *DHPS* on one allele and a more severe mutation on the other. The combination of missense and hypomorphic variants reduces enzyme activity and impairs hypusination of eukaryotic translation initiation factor 5A^4^. Functional studies using patient-derived lymphoblast cell lines and model organisms (e.g., yeast, zebrafish, mice) show that these mutations decrease eIF5A hypusination, impairing mRNA translation^4–8^. Depending on the organ in which DHPS activity is reduced, translation of specific targets is affected; in the brain, severe neuronal loss or dysfunction occurs with genetic deletion of *Dhps* in mice^8^. These model systems demonstrate that partial loss of DHPS activity can disrupt essential developmental processes, and the brain is particularly vulnerable to reduced DHPS activity^4,9^.

Studies reveal broader cellular effects of impaired DHPS function, including alterations in oxidative stress, mitochondrial function, and autophagy^4,10^. Thus, defective mRNA translation affects the brain and cellular systems (e.g., proteostasis, metabolism), contributing to the effects of DHPS deficiency. Although no disease-modifying treatments are available, emerging research points to innovative therapeutic strategies (e.g., small-molecule modulation of hypusination). Understanding the spectrum of clinical, molecular, and biochemical features of DHPS deficiency will enable the development of targeted treatments and uncover the roles of hypusination in human health and disease.

Here, we generated human brain organoids from patient-derived induced pluripotent stem cells (iPSCs) and their CRISPR-corrected isogenic controls. These iPSCs were used to produce semi-guided brain cortical organoids^11,12^. Significantly decreased organoid growth was observed in the mutants compared to controls, highlighting DHPS’s role in neurodevelopment. Next, we developed a gene therapy approach by inserting the *DHPS* cDNA into an adeno-associated virus (AAV) serotype 9 to deliver the correct gene copy into mutant organoids. The gene therapy strategy successfully restored hypusination in human brain cells. We assessed our gene therapy in a mouse model of brain-specific DHPS loss and successfully restored growth, metabolic health, and reduced premature death. Our results provide preliminary evidence that a gene therapy strategy may benefit individuals with DHPS deficiency.

## Results

### Human brain organoids with DHPS deficiency

**Figure 1:**
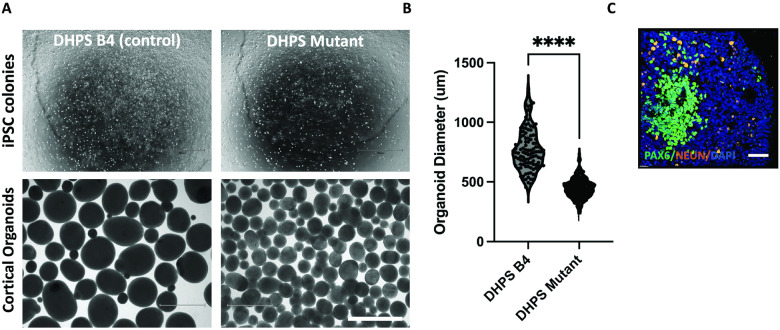
A human brain organoid model for DHPS deficiency. **A**, Bright field images of human iPSCs (top) and derived brain organoids at 1 month (bottom). Scale bar = 50 mm. **B**, Distribution of cortical organoid diameters, comparing control (B4) and mutant isogenic cells (n = 100). **C**. Cross section of a 1-month-old brain organoid showing a typical rosette structure surrounded by proliferative PAX6-positive cells and more differentiated neurons (NeuN-positive cells). Statistical analysis was performed using one-way ANOVA followed by Tukey’s multiple comparisons test. *p < 0.05; **p< 0.01; ***p < 0.001; ****p < 0.0001.

To generate human brain cells lacking DHPS activity, we used induced pluripotent stem cells (iPSCs), reprogrammed from a donor patient carrying two *DHPS* mutations (**Fig. S1A**). An isogenic control line was generated by correcting these mutations using genome-editing technologies (**Fig. S1B**). The mutant iPSC is called DHPS-mutant, and the control line is named DHPS B4. These iPSCs were expanded under similar conditions and used to generate human cortical organoids using a semi-guided protocol^11,12^. While no differences in growth were observed at the pluripotent stage (Fig. 1A), the cortical organoids derived from the mutant showed a significant size reduction (diameter) compared to controls (Fig. 1A, B). Cortical organoids from both genotypes formed rosettes containing proliferative cells (PAX6+) and markers of corticogenesis (NEUN+) (Fig. 1C).

### A gene therapy strategy to deliver the human DHPS cDNA to brain cells

**Figure 2:**
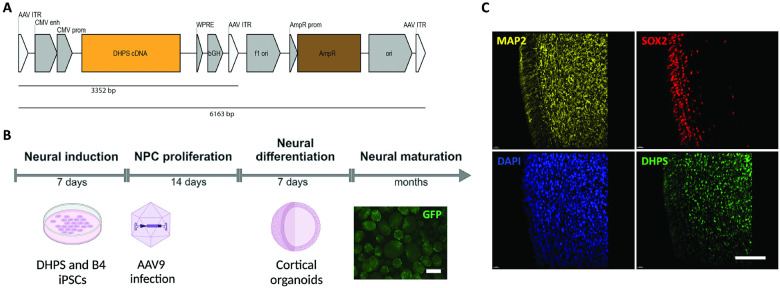
A gene therapy strategy for DHPS deficiency. **A**, Schematic of the AAV vector carrying the DHPS cDNA under the control of the CMV promoter. **B**, Timeline of the cortical organoid infection with the AAV particles. The AAV-GFP was performed in parallel for visualization. Scale bar = 750 mm. **C**, Immunostainings of DHPS mutant brain cortical organoids 30 days after AAV infection showing restoration of DHPS expression. Scale bar = 50 mm.

We cloned the human *DHPS* cDNA into an AAV9 backbone driven by the CMV promoter (Fig. 2A). Viral particles were produced and used to infect the DHPS-mutant cortical organoids during the proliferation phase (Fig. 2B). We collected total protein extracts for downstream analyses at 36- and 60-days post-infection. We confirmed DHPS enzyme expression in mutant cells at levels similar to those in control organoids (Fig. 2C and S2A). Next, the total protein extract was used to verify the transgene’s enzymatic activity in human brain cells using the deoxyhypusine hydroxylase assay (**Fig. S2B**).

### Restoring DHPS expression in vivo in a mouse model of brain-specific loss of DHPS

**Figure 3:**
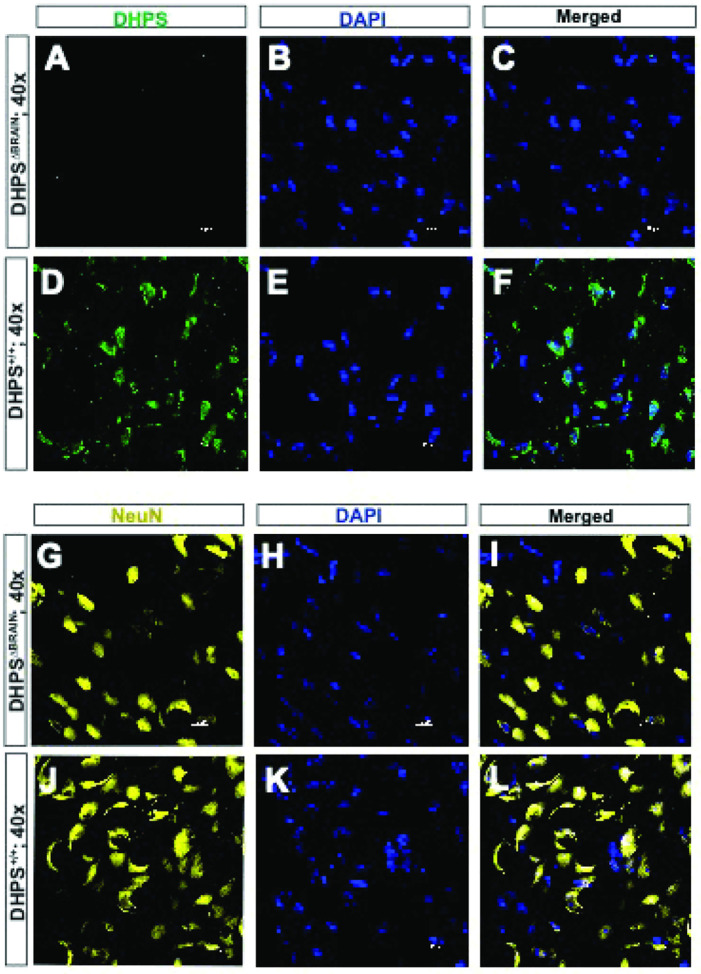
DHPS^ΔBRAIN^ mice show loss of DHPS protein, but unchanged mature neuron abundance. Immunofluorescent staining for DHPS (green) and nuclei (DAPI, blue) of 3-week-old brain tissue from (**A-C**) DHPS^ΔBRAIN^ and (**D-F**) Control mice show reduced DHPS levels in mutant mice. Immunofluorescent staining of mature neurons (NeuN, yellow) and nuclei (DAPI, blue) for 3-week-old (**G-I**) DHPS^ΔBRAIN^ and (**J-L**) control mice show no change in mature neuron abundance. Scale bar = 10 mm.

**Figure 4:**
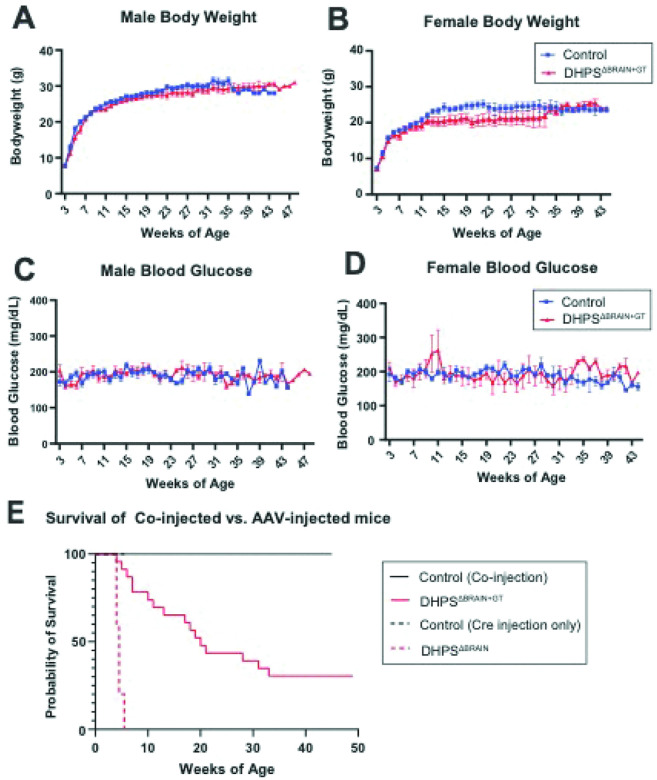
Gene therapy injection does not affect body weight or blood glucose levels, but it improves long-term survival. Body weight in (**A**) male and (**B**) female DHPS^ΔBRAIN+GT^ and control *(Dhps^LoxP/+^;R26R^TOMATO^)* mice from weaning (3 weeks) to 48 weeks (n=11–12/group). blood glucose measurements in (**C**) male and (**D**) female DHPS^ΔBRAIN+GT^ and control mice from weaning to 48 weeks (n=10–14/group). (**E**) Survival curve showing the length of survival (in weeks-of-age) of DHPS^ΔBRAIN^, DHPS^ΔBRAIN+GT^, control mice co-injected with the DHPS overexpression and knockdown viruses (labeled as “control co-injection”), and control mice injected with only the Cre virus (labeled as “control cre injection only”).

Compared with controls, DHPS^ΔBRAIN^ mouse model mutants showed reduced growth after 3 weeks-of-age, premature death by 6 weeks-of-age, and intermittent seizures^4^. We used this mouse model to investigate whether restoring expression through gene therapy could rescue the observed phenotypes. To confirm that our mouse model represents a loss of DHPS in the brain, we performed immunofluorescent staining of DHPS and observed successful DHPS knockdown in DHPS^ΔBRAIN^ mouse brains (Fig. 3A–F). Mature neuron abundance, visualized by NeuN staining, was similar in the DHPS^ΔBRAIN^ mice and controls (Fig. 3G–L). These results were obtained from male and female mouse tissue. To determine if *Dhps* re-expression could rescue the phenotypes of reduced animal growth and early lethality by 6 weeks of age, we co-injected P0 mice with equal titers of scAAV2/9-CMV-Cre and the *Dhps* overexpression virus AAV9-CMV-DHPS to generate the DHPS^ΔBRAIN+GT^ mice. Weekly measurements of body weight (Fig. 4A–B) and blood glucose (Fig. 4C–D) in male and female mice were similar in DHPS^ΔBRAIN+GT^ and control animals, indicating that growth and metabolic health remained stable with age. The survival of DHPS^ΔBRAIN+GT^ mice was significantly improved compared with DHPS^ΔBRAIN^ mice (Fig. 4E). We monitored 47 mice (23 males and 24 females) for survival; 6 DHPS^ΔBRAIN+GT^ mice survived past 43 weeks, with 1 surviving to 48 weeks. Among the long-term survivors of both groups, seizures were observed in 6 DHPS^ΔBRAIN+GT^ mice and 2 control mice near the end of their lifespan. This data demonstrates that re-expression of DHPS in a mouse model of DHPS loss in the brain restores overall growth and metabolic health and prolongs survival.

## Discussion

Using genome-editing techniques, we generated the first human brain organoid model of DHPS deficiency in isogenic iPSCs. Brain organoids are three-dimensional, stem cell–derived neural tissues that recapitulate key aspects of early human brain development, including cell-type diversity, spatial organization, and developmental trajectories, thereby providing a uniquely human platform for modeling neurodevelopmental disorders *in vitro*^13^. Our model reproduces the developmental deficiency associated with DHPS loss, as evidenced by reduced organoid size. Importantly, the use of brain organoids offers translational advantages, as they enable testing of human-specific therapeutic strategies in a controlled context; for example, recent work in Pitt-Hopkins syndrome demonstrated the development and validation of a gene therapy in patient-derived brain organoids prior to *in vivo* application^14^. In our study, we used an AAV vector to restore DHPS expression in human brain cells without signs of toxicity, supporting the feasibility of gene replacement approaches in a human neural context for this condition.

*In vivo*, restoring DHPS expression in mouse brains with complete DHPS loss extended survival and rescued growth impairments, suggesting that reintroducing functional DHPS early in development may ameliorate or reverse some clinical manifestations of DHPS deficiency. Although lifespan data for individuals with DHPS deficiency are lacking, these findings underscore the therapeutic potential of DHPS restoration. Variability in survival outcomes among treated mice may reflect technical differences in viral delivery efficiency, including injection accuracy and ventricular biodistribution. The growth reduction observed in the complete loss model may specifically result from the absence of DHPS, as clinical phenotypes related to weight or metabolism are not consistently reported among affected individuals. This interpretation is supported by a pancreas-specific DHPS knockout model^7^, which exhibits reduced growth and early lethality, paralleling the DHPS^ΔBRAIN^ phenotype and reinforcing DHPS’s role in systemic growth regulation.

Despite these promising findings, both model systems present important limitations. While brain organoids capture human-specific developmental features, they lack vascularization, immune components, and long-range connectivity, and they model primarily early developmental stages, which may limit their ability to predict long-term therapeutic outcomes^13^. Conversely, mouse models provide whole-organism context and enable assessment of survival and systemic phenotypes, but they do not fully recapitulate human neurodevelopment or the genetic nuances of DHPS deficiency, as patient mutations typically result in partial (~ 20%) residual enzyme activity rather than complete loss. Thus, while genetic deletion of DHPS offers a robust platform for evaluating therapeutic strategies, it does not fully capture the spectrum or subtlety of the human condition. Future studies using mouse models that more accurately reflect the hypomorphic nature of patient mutations, in combination with advanced human organoid systems, will be essential to more precisely assess the efficacy, timing, and durability of DHPS re-expression and other targeted interventions.

## Materials and Methods

### iPSC lines and cell culture

For this work, we used a patient-derived iPSC line carrying two DHPS mutations in exons 4 and 8 (c.518 A > G/+; c.1014 + 1 G > A/+) and the respective double CRISPR-corrected isogenic iPSC line as controls). Briefly, we identified a gRNA sequence (5’-CAAGATAAGCGCTGGCTGGG-3’) with 5’-TGG-3’ PAM, capable of positioning the DHPS c.1014 + 1 G > A mutation within the 3–9 editing window of ABEmax at position A5. Patient-derived iPSCs were co-transfected with plasmids encoding gRNA under the expression of the U6 promoter and ABEmax-T2A-eGFP under the expression of the CMV promoter using the Lonza P3 nucleofection system with protocol CA-137. 72-hours post-nucleofection, cells were collected in a single cell suspension using accutase and stained with DAPI for live/dead detection during FACS. Nucleofected cells were single cell sorted based on GFP expression conferred through plasmid uptake and DAPI live/dead selection on the SY3200 HAPS sorter. Cells were clonally expanded and Sanger sequenced at the DHPS c.1014 + 1 locus to identify corrected clones. Corrected clones were subsequently CRISPR edited at the c.518 A > G locus to generate double CRISPR-corrected isogenic iPSCs. We cloned the gRNA (5’-AACTTGCAGTAATTCTCACT-3’) with 5’-GGG-3’ PAM under the expression of the U6 promoter into plasmid px458 using Golden Gate assembly. Single corrected iPSCs were transfected with the c.518 A > G targeting gRNA encoding px458 plasmid along with a 180-bp single-stranded oligodeoxynucleotide donor encoding the wild-type DHPS c.518 A sequence for templated homology directed repair. Transfected cells were sorted and genotyped as mentioned above. As previously described^15–18^, we maintained hiPSC colonies on Matrigel-coated (BD Biosciences, San Jose, CA, USA) 6-cm plates. We kept the cells in mTeSR plus (StemCell Technologies, Vancouver, Canada), changing the media every other day. We exclusively utilized cell cultures that tested negative for mycoplasma contamination. The experiments adhered to the ethical principles outlined in the WMA Declaration of Helsinki and the Belmont Report, as established by the U.S. Department of Health and Human Services. The study received approval from the UCSD IRB/ESCRO committee under protocol 141223.

### Generation of brain organoids

We generated cortical organoids following previously described semi-guided protocols^11,12^. Briefly, we cultured hiPSCs for approximately 6 days and then dissociated them using a 1:1 Accutase (Life Technologies): PBS solution. Subsequently, we plated the cells into a 6-well plate (4×10^6^ cells/well) in mTeSR plus supplemented with 10 μM SB431542 (SB; Stemgent, Cambridge, MA, USA), 1 μM Dorsomorphin (Dorso; R&D Systems, Minneapolis, MN, USA), and 5 μM Y-27632 (EMD-Millipore, Burlington, MA, USA). The cells were then cultured in shaker suspension (95 rpm at 37°C). We fed them mTeSR-plus supplemented with 10 μM SB and 1 μM Dorso for three days to nourish the emerging spheres. Subsequently, the medium was changed to Media1 [Neurobasal (Life Technologies), 1x Glutamax (Life Technologies), 2% Gem21-NeuroPlex (Gem21; Gemini Bio-Products, Sacramento, CA, USA), 1% N2-NeuroPlex (N2; Gemini Bio-Products), 1% non-essential amino acids (NEAA; Life Technologies), 1% penicillin/streptomycin (P/S; Life Technologies), 10 μM SB, and 1 μM Dorso] and cultivated every other day for six days. Afterward, we started the proliferation stage and used Media2 (Neurobasal, 1x Glutamax, 2% Gem21, 1% NEAA, and 1% P/S) supplemented with 20 ng/mL FGF-2 (Life Technologies) daily for seven days. After the first-step proliferation stage, we followed a second stage of cell proliferation using Media2 supplemented with 20 ng/mL each of FGF-2 and EGF (PeproTech, Rocky Hill, NJ, USA) every other day for 6 days. For the subsequent six days, we cultured the organoids in Media2 supplemented with 10 ng/mL each of BDNF, GDNF, and NT-3 (all PeproTech), 200 μM L-ascorbic acid (Sigma-Aldrich, St. Louis, MO, USA), and 1 mM dibutyryl-cAMP (Sigma-Aldrich) every other day. Finally, we maintained the cortical organoids until day 45 in Media2 without supplementation.

### AAV Infection in Human Brain Organoids

On day 11 of the cortical brain organoid protocol described above, which marks the beginning of the NPC proliferation phase, infections were performed in 30–50 organoids per condition. This was done by adding 1×10^10 vg/well of AAV9-DHPS-WPRE-bGH, an adeno-associated virus serotype 9 (AAV9) that contains the full-length DHPS protein under the control of a CMV promoter, or a vector where eGFP, both obtained from VectorBuilder, replaces the DHPS sequence. The diameter of the organoids was measured using an automated colony counter (GelCount, Oxford Optronix) on days 0, 15, and 30 after AAV9 infection. Statistical comparisons were performed using an ordinary one-way ANOVA test followed by Tukey’s Honestly Significant Difference (HSD) post-hoc test, with a significance threshold of p = 0.05. P-values are indicated by asterisks in the figures for significance levels: p < 0.05 (*), p < 0.01 (**), p < 0.001 (***), or p < 0.0001 (****). The infected organoids were harvested for cryosectioning at day 30 postinfection for further analysis.

### Immunofluorescence stainings

We conducted immunofluorescence staining following previously described methods^17–21^. We fixed the cortical brain organoids in 4% paraformaldehyde for 4 hours, then transferred them to 30% sucrose and embedded them in O.C.T. (Sakura, Tokyo, Japan). Subsequently, we sectioned the organoids at 20 μm using a cryostat. The resulting organoid sections on slides were air-dried, permeabilized, and blocked using 0.1% Triton X-100 and 3% bovine serum albumin (BSA) in phosphate-buffered saline (PBS). We applied primary antibodies (rabbit anti-DHPS, ProteinTech #11184–1-AP 1:100; chicken anti-GFAP, Abcam #4674, 1:500; guinea pig anti-NeuN, Synaptic Systems #266004, 1:200) diluted in blocking buffer and incubated them overnight at 4°C. After washing the slides three times with PBS (each for five minutes), we incubated them with secondary antibodies (Alexa Fluor 555, 647, and 488, Life Technologies) diluted 1:1000 in a blocking buffer. We stained the cell nuclei with DAPI (diluted 1:10,000 in PBS) for 5 minutes. Finally, we mounted the slides with ProLong Gold anti-fade mounting medium (Life Technologies) and captured and analyzed the images using a Z1 Axio Observer Apotome fluorescence microscope (Zeiss, Oberkochen, Germany).

### Proliferation and apoptosis analysis

For cell cycle analysis by flow cytometry, 10–15 organoids aged 15 days were collected and dissociated following the protocol described below. Briefly, the organoids were transferred to a new 6-well plate, where the medium was aspirated and washed with an EDTA/PBS (1:1000) solution. After removing EDTA/PBS, 10X TrypLE (Thermo Fisher, no. A1217701) was added, and the spheres were incubated at 37°C on a shaker set to 95 rpm for 1 hour. The organoids were then transferred to a new tube with blocking solution, consisting of DMEM/F12 (Gibco) supplemented with 1 μM CaCl_2_, 0.5% bovine serum albumin (BSA), and 400 U/mL DNase. After complete dissociation of the organoids by up-and-down pipetting, the cells were centrifuged for 5 minutes at 0.4 x g, resuspended in blocking solution with DNAse, and kept in a 37°C water bath for 5 minutes to remove clumps, repeated twice before cell counting. Three hundred thousand individual cells were then centrifuged for 5 minutes at 0.4 x g and washed with 0.5% BSA in 1X PBS prior to flow cytometry analysis. For DAPI staining, the Transcription Factor Buffer Kit (BD Pharmingen, no. 562574) was used according to the manufacturer’s instructions. Cells were analyzed using a FACSCanto II (BD Biosciences) flow cytometer and FlowJo software.

### Deoxyhypusine hydroxylase assay

We performed the deoxyhypusine hydroxylase assay as previously described^22^. Briefly, deoxyhypusine hydroxylase (DOHH) activity was measured by evaluating the conversion of deoxyhypusine to hypusine on eIF5A in human cells. Cell lysates containing deoxyhypusine-eIF5A were incubated in a reaction mixture supplemented with NAD^+^ and Fe^2+^, which are essential cofactors for DOHH catalysis. After incubation, the level of hypusine-eIF5A was quantified using high-performance liquid chromatography (HPLC) or liquid chromatography–mass spectrometry (LC–MS), enabling separation and detection of hypusine and deoxyhypusine residues. In some experiments, hypusination levels were also confirmed by Western blotting using antibodies specific for hypusinated eIF5A. This assay allowed assessment of DOHH enzymatic activity and its response to different experimental conditions.

### Proliferation and apoptosis analysis

For cell cycle analysis by flow cytometry, 10–15 organoids aged 15 days were collected and dissociated following the protocol outlined below. Briefly, the organoids were transferred to a new 6-well plate, where the medium was aspirated, and the plate was washed with an EDTA/PBS (1:1000) solution. After removing the EDTA/PBS, 10X TrypLE (Thermo Fisher, no. A1217701) was added, and the spheres were incubated at 37°C on a shaker set to 95 rpm for 1 hour. The organoids were then transferred to a new tube containing blocking solution, which was made of DMEM/F12 (Gibco) supplemented with 1 μM CaCl_2_, 0.5% bovine serum albumin (BSA), and 400 U/mL DNase. After fully dissociating the organoids with up-and-down pipetting, the cells were centrifuged for 5 minutes at 0.4 x g, resuspended in blocking solution with DNase, and kept in a 37°C water bath for 5 minutes to remove clumps. This process was repeated twice before cell counting. Three hundred thousand individual cells were then centrifuged for 5 minutes at 0.4 x g, washed with 0.5% BSA in 1X PBS, and prepared for flow cytometry analysis. For DAPI staining, the Transcription Factor Buffer Kit (BD Pharmingen, no. 562574) was used according to the manufacturer’s instructions. Cells were analyzed using a FACSCanto II (BD Biosciences) flow cytometer and FlowJo software.

### Animal studies

Animal studies were conducted at Indiana University Indianapolis, where animals were maintained under a protocol approved by the Indiana University Indianapolis School of Science Institutional Animal Care and Use Committee. Since a genetic null for *Dhps* in mice is embryonic lethal^8,23^, a mouse model with a conditional genetic deletion of *Dhps* in the brain was previously generated, validated, and characterized^4,8^. Therefore, we used this model to investigate whether overexpression of *Dhps* in the brain via gene therapy could rescue the phenotype. Mice carrying both the *Dhps*^*LoxP*^ allele (B6.Cg-Dhps^tm1.1Mirm/J^)^24^ and the *R26R*^*Tomato*^ reporter allele (B6.Cg-Gt(ROSA)26Sor^tm14(CAG-tdTomato)Hze/J^)^25^ were maintained on a mixed genetic background. Timed matings were performed, with noon on the day a vaginal plug was observed marking embryonic day (E) 0.5. On the day of birth, postnatal day (P) 0, all pups in each litter were anesthetized and received an intraventricular injection of 2.5 μL of the following solution: 0.5 μL of self-complementary adeno-associated virus 2/9 (scAAV2/9) carrying a CMV promoter that drives Cre recombinase expression (scAAV2/9-CMV-Cre; 1.35e + 13 vg/mL; University of Iowa Viral Vector Core, no. AAV3558), 0.5 μL of AAV9-CMV-DHPS (1.35e + 13 vg/mL; as described in the AAV-mediated DHPS overexpression section below), and 2 μL of trypan blue (Thermo Fisher Scientific, no. 12-250-061). The intracerebroventricular injection promoted viral migration between brain hemispheres, enabling infection of all brain cell types. Cre recombinase expression induced the deletion of *Dhps* in animals carrying two *Dhps*^*LoxP*^ alleles, leading to the loss of DHPS protein in those cells. Additionally, infected cells expressed the Cre-dependent *R26R*^*Tomato*^ reporter, enabling the identification of all virally infected cells via Tomato expression. After the injection, pups were tattooed (Thermo Fisher Scientific, no. NC9665212) for identification, and a tail biopsy was performed to obtain DNA for genotyping using previously described primers^24,25^. Experimental animals are designated DHPS^ΔBRAIN^ for *Dhps*^*LoxP/LoxP*^; *R26R*^*Tomato animals*^ injected with the Cre virus only, and DHPS^ΔBRAIN+GT^ for *Dhps*^*LoxP/LoxP*^; *R26R*^*Tomato*^ animals co-injected with the Cre and gene therapy viruses. All pups within a litter were injected identically, ensuring that all genotypes (wildtype, *Dhps*^*LoxP/+*^, *Dhps*^*LoxP/LoxP*^) received the same dosage of each virus. Once animals reached weaning age (3 weeks), their weight was measured using a digital scale (Thermo Fisher Scientific), and blood glucose levels were checked with an AlphaTrak2 glucose monitor and test strips (Thermo Fisher Scientific). Animal weight and blood glucose were recorded weekly, and any adverse health events, such as seizures, were documented. For each cohort, survival was tracked until death (or up to 48 weeks) for all experimental animals and littermate controls.

### Tissue preservation and immunofluorescent staining of mouse tissue

To preserve brain tissue, animals were anesthetized with avertin, then perfused transcardially with 5 mL of 4% paraformaldehyde (Acros Organics), followed by 5 mL of PBS. After perfusion, mouse brains were fixed in 4% paraformaldehyde (Acros Organics), cryopreserved in 30% sucrose (ThermoFisher Scientific), and embedded in OCT (Thermo Fisher Scientific). Brains were cryo-sectioned (15 μM, coronal) using a CM1950 Cryostat (Leica). Slides were permeabilized in PBST (0.1% Triton-X 100 (Fisher Scientific) in PBS (Fisher Scientific)). Slides were blocked in 5% Normal Donkey Serum (Sigma-Aldrich) in PBS. After blocking, primary antibodies were diluted in blocking solution at the following concentrations: rabbit anti-NeuN (Abcam, no. ab177487, 1:1000), rabbit anti-DHPS (ProteinTech, no. 1184, 1:100). Slides were incubated in primary antibody solutions overnight at 4°C. Slides were then washed in PBST followed by incubation at room temperature for 2 hours with fluorescently labeled secondary antibodies (Alexa Fluor-647, Fisher Scientific), diluted 1:500 in blocking solution. Finally, slides were stained with DAPI (1:1000, Sigma-Aldrich) to identify nuclei, washed in PBS, and mounted using Fluoro-Gel mounting media (Fisher Scientific).

### Statistical analysis

We performed statistical analyses using GraphPad Prism v9 software (GraphPad Software, La Jolla, CA). Sample sizes were determined based on prior publications from our laboratory and other relevant sources. Experiment-specific details about samples and cell lines are provided in the figure legends. Samples were randomized, allocated, and evaluated according to the treatment. Outliers in other experiments were identified and excluded based on GraphPad criteria. Continuous variables are shown as mean ± standard error of the mean, and 95% confidence intervals were calculated under the assumption of normality. Normality was assessed visually or using GraphPad analysis, and variance was accounted for in all analyses. For group comparisons, the means of continuous variables were analyzed using appropriate statistical tests, such as the unpaired Student’s t-test, one-way or two-way analysis of variance (ANOVA), or the Mann-Whitney U test for nonparametric data. Two-sided tests were used, with a significance level (α) of 0.05.

## Supplementary Material

This is a list of supplementary files associated with this preprint. Click to download.

- SupFigure1SantoUCSD.tif
- SupFigure2SantoUCSD.tif
- SupWB.tif

## Data Availability

Microscopy images from this study were not uploaded to public repositories due to the inclusion of sensitive information related to human patients. However, these data can be made available upon reasonable request to the corresponding authors, subject to approval by the institutions’ Ethics Committees responsible for collecting or maintaining the patient’s biological samples and cells. Source data are included in this manuscript.

## References

[1] Ganapathi M, Recessive Rare Variants in Deoxyhypusine Synthase, an Enzyme Involved in the Synthesis of Hypusine, Are Associated with a Neurodevelopmental Disorder. Am J Hum Genet. 2019;104:287–98. 10.1016/j.ajhg.2018.12.017. 30661771 PMC6369575

[2] Nguyen S, Deoxyhypusine Modification of Eukaryotic Translation Initiation Factor 5A (eIF5A) Is Essential for Trypanosoma brucei Growth and for Expression of Polyprolyl-containing Proteins. J Biol Chem. 2015;290:19987–98. 10.1074/jbc.M115.656785. 26082486 PMC4528157

[3] Templin AT, Maier B, Nishiki Y, Tersey SA, Mirmira RG. Deoxyhypusine synthase haploinsufficiency attenuates acute cytokine signaling. Cell cycle (Georgetown Tex. 2011;10:1043–9. 10.4161/cc.10.7.15206. 21389784 PMC3100881

[4] Padgett LR, Deoxyhypusine synthase mutations alter the post-translational modification of eukaryotic initiation factor 5A resulting in impaired human and mouse neural homeostasis. HGG Adv. 2023;4:100206. 10.1016/j.xhgg.2023.100206. 37333770 PMC10275725

[5] Shojaeinia E, Deoxyhypusine synthase deficiency syndrome zebrafish model: aberrant morphology, epileptiform activity, and reduced arborization of inhibitory interneurons. Mol Brain. 2024;17:68. 10.1186/s13041-024-01139-w. 39334388 PMC11429087

[6] Connors CT, A Translational Regulatory Mechanism Mediated by Hypusinated Eukaryotic Initiation Factor 5A Facilitates beta-Cell Identity and Function. Diabetes. 2024;73:461–73. 10.2337/db23-0148. 38055903 PMC10882153

[7] Padgett LR, Deoxyhypusine synthase, an essential enzyme for hypusine biosynthesis, is required for proper exocrine pancreas development. FASEB J. 2021;35:e21473. 10.1096/fj.201903177R. 33811703 PMC8034418

[8] Kar RK, Neuron-specific ablation of eIF5A or deoxyhypusine synthase leads to impairments in growth, viability, neurodevelopment, and cognitive functions in mice. J Biol Chem. 2021;297:101333. 10.1016/j.jbc.2021.101333. 34688659 PMC8605248

[9] Pallmann N, Biological Relevance and Therapeutic Potential of the Hypusine Modification System. J Biol Chem. 2015;290:18343–60. 10.1074/jbc.M115.664490. 26037925 PMC4513096

[10] Anderson-Baucum E Deoxyhypusine synthase promotes a pro-inflammatory macrophage phenotype. Cell Metab 33, 1883–1893 e1887 (2021). 10.1016/j.cmet.2021.08.003 34496231 PMC8432737

[11] Fitzgerald MQ, Generation of ‘semi-guided’ cortical organoids with complex neural oscillations. Nat Protoc. 2024. 10.1038/s41596-024-00994-0. PMC11380594 38702386

[12] Trujillo CA, Complex Oscillatory Waves Emerging from Cortical Organoids Model Early Human Brain Network Development. Cell Stem Cell. 2019. 10.1016/j.stem.2019.08.002. PMC6778040 31474560

[13] Tenreiro MF, Muotri AR. Reconstructing human corticogenesis: Insights from cerebral organoids into neurodevelopment and disease modeling. Dev Cell. 2026;61:720–43. 10.1016/j.devcel.2026.02.018. 41875881 PMC13014016

[14] Papes F, Transcription Factor 4 loss-of-function is associated with deficits in progenitor proliferation and cortical neuron content. Nat Commun. 2022;13:2387. 10.1038/s41467-022-29942-w. 35501322 PMC9061776

[15] de Souza JS, IGF1 neuronal response in the absence of MECP2 is dependent on TRalpha 3. Hum Mol Genet. 2017;26:270–81. 10.1093/hmg/ddw384. 28007906 PMC6075524

[16] de Souza JS, Altered Gene Expression of Thyroid Hormone Transporters and Deiodinases in iPS MeCP2-Knockout Cells-Derived Neurons. Mol Neurobiol. 2019;56:8277–95. 10.1007/s12035-019-01645-2. 31214863

[17] Mesci P, SARS-CoV-2 infects human brain organoids causing cell death and loss of synapses that can be rescued by treatment with Sofosbuvir. PLoS Biol. 2022;20:e3001845. 10.1371/journal.pbio.3001845. 36327326 PMC9632769

[18] Papes F, Transcription Factor 4 loss-of-function is associated with deficits in progenitor proliferation and cortical neuron content. Nat Commun. 2022;13:2387. 10.1038/s41467-022-29942-w. 35501322 PMC9061776

[19] Trujillo CA, Pharmacological reversal of synaptic and network pathology in human MECP2-KO neurons and cortical organoids. EMBO Mol Med. 2021;13:e12523. 10.15252/emmm.202012523. 33501759 PMC7799367

[20] Trujillo CA Complex Oscillatory Waves Emerging from Cortical Organoids Model Early Human Brain Network Development. Cell Stem Cell 25, 558–569.e557 (2019). 10.1016/j.stem.2019.08.002 31474560 PMC6778040

[21] Trujillo CA, Reintroduction of the archaic variant of. Science. 2021;371. 10.1126/science.aax2537. PMC8006534 33574182

[22] Park JH, Wolff EC, Park MH. Assay of deoxyhypusine hydroxylase activity. Methods Mol Biol. 2011;720:207–16. 10.1007/978-1-61779-034-8_13. 21318876 PMC3178843

[23] Nishimura K, Lee SB, Park JH, Park MH. Essential role of eIF5A-1 and deoxyhypusine synthase in mouse embryonic development. Amino Acids. 2012;42:703–10. 10.1007/s00726-011-0986-z. 21850436 PMC3220921

[24] Levasseur EM, Hypusine biosynthesis in beta cells links polyamine metabolism to facultative cellular proliferation to maintain glucose homeostasis. Sci Signal. 2019;12. 10.1126/scisignal.aax0715. PMC7202401 31796630

[25] Madisen L, A robust and high-throughput Cre reporting and characterization system for the whole mouse brain. Nat Neurosci. 2010;13:133–40. 10.1038/nn.2467. 20023653 PMC2840225

